# Delineation and detection of breast cancer using novel label-free fluorescence

**DOI:** 10.1186/s12880-023-01095-2

**Published:** 2023-09-16

**Authors:** Alaaeldin Mahmoud, Yasser H. El-Sharkawy

**Affiliations:** Optoelectronics and automatic control systems department, Military Technical College, Kobry El-Kobba, Cairo, Egypt

**Keywords:** Optical diagnosis, Hyperspectral imaging, Blue Laser, Fluorescence, K-mean clustering, Breast tumor

## Abstract

**Background:**

Accurate diagnosis of breast cancer (BC) plays a crucial role in clinical pathology analysis and ensuring precise surgical margins to prevent recurrence.

**Methods:**

Laser-induced fluorescence (LIF) technology offers high sensitivity to tissue biochemistry, making it a potential tool for noninvasive BC identification. In this study, we utilized hyperspectral (HS) imaging data of stimulated BC specimens to detect malignancies based on altered fluorescence characteristics compared to normal tissue. Initially, we employed a HS camera and broadband spectrum light to assess the absorbance of BC samples. Notably, significant absorbance differences were observed in the 440–460 nm wavelength range. Subsequently, we developed a specialized LIF system for BC detection, utilizing a low-power blue laser source at 450 nm wavelength for ten BC samples.

**Results:**

Our findings revealed that the fluorescence distribution of breast specimens, which carries molecular-scale structural information, serves as an effective marker for identifying breast tumors. Specifically, the emission at 561 nm exhibited the greatest variation in fluorescence signal intensity for both tumor and normal tissue, serving as an optical predictive biomarker. To enhance BC identification, we propose an advanced image classification technique that combines image segmentation using contour mapping and K-means clustering (K-mc, K = 8) for HS emission image data analysis.

**Conclusions:**

This exploratory work presents a potential avenue for improving "in-vivo" disease characterization using optical technology, specifically our LIF technique combined with the advanced K-mc approach, facilitating early tumor diagnosis in BC.

**Supplementary Information:**

The online version contains supplementary material available at 10.1186/s12880-023-01095-2.

## Introduction

BC is a prevalent and life-threatening disease affecting women worldwide. Early diagnosis is crucial for improving survival rates among individuals with BC [1–3]. Unfortunately, more than 8% of women are expected to develop this malignancy at some point in their lives [4]. BC ranks as the second most common tumor in the United States [5]. It is characterized by the uncontrolled proliferation of breast cells, with invasive ductal carcinoma (IDC) and invasive lobular carcinoma (ILC) being the most prevalent types [6, 7]. The female breast primarily comprises adipose, glandular, and connective tissues, as well as lymphatic and blood vessels. It consists of several lobes, each connected to different lactiferous ducts [8–11]. Additionally, the breast stroma includes stromal cells and the cellular membrane (CM), composed of various proteins, water molecules, and polysaccharides [12]. The CM plays a critical role in the interaction between stromal cells and the transmission of biochemical and biophysical signals. Changes in CM structure, along with inflammatory cell infiltration and fibroblast separation, have been associated with BC development [13]. Early detection of BC is vital for improved treatment outcomes and reduced mortality rates [14]. Mammography and computed tomography are standard diagnostic procedures used to identify potentially cancerous or benign masses or lumps in breast tissue. However, these methods have limitations such as inaccuracy, high costs, exposure to radiation, and patient discomfort during the examination [15]. Additionally, there is a lack of clinically accessible intraoperative resection edge methods, making breast medical treatments challenging. Consequently, up to 37% of identified BC cases are found at the resection edge [16–19]. Moreover, previous studies have linked tumor recurrence and metastasis to the presence of positive resection margins [20]. Various approaches have been employed to assess resection edges during breast-conserving surgeries (BCS) to minimize the presence of tumor-positive margins [21–23]. While these methods have gained popularity and proven beneficial, they also have limitations. Physical examinations may struggle to detect small cancers, and mammography often produces false-positive results [24]. Optical spectroscopy and imaging techniques for diagnosing breast tumors are still in the early stages of development. However, the need for sensitive and early cancer diagnosis, coupled with technological advancements, has played a significant role in driving research in this field.

Fluorescence spectroscopy [25–28] has been used to many various sorts of materials during the last few decades, ranging from individual biochemical species to organs of living persons. It has been used for practically every form of BC, both in-/ ex-vivo, and has shown advantages over other light-based approaches in terms of sensitivity, speed, and safety. NADH, FAD, collagen, elastin, lipids, aromatic acids, and porphyrins are the most significant endogenous fluorophores that glow in the UV/VIS spectral region [29]. Fluorescence in heterogeneous systems, such as tissue, is caused by all of the fluorophores present. Such alterations occur during malignant cell transformations, and they are reflected in the fluorescence characteristics of the BC tissue. Fluorescent approaches are now capable of detecting and characterizing metabolic and pathological alterations in precancerous and cancerous tissues as compared to normal tissue [30, 31]. Fluorescence spectroscopy for BC detection typically involves the acquisition of fluorescence signals across a range of wavelengths using specialized detectors [32, 33]. These signals can be processed and analyzed to extract relevant information about the tissue's biochemical and morphological properties. Differences in fluorescence intensity, emission spectra, or fluorescence lifetime between cancerous and non-cancerous regions can be indicative of the presence of BC [34–36]. Comparing fluorescence spectroscopy with other imaging techniques, such as mammography or magnetic resonance imaging (MRI), reveals its unique strengths. Mammography provides excellent spatial resolution but is limited in its ability to distinguish between benign and malignant lesions accurately [37]. MRI offers superior soft tissue contrast but is more expensive and time-consuming [38]. On the other hand, the non-ionizing nature of fluorescence spectroscopy makes it a safer alternative to ionizing radiation-based imaging modalities like mammography and computed tomography [39]. It offers the potential for repeated examinations without excessive radiation exposure. Moreover, fluorescence spectroscopy can provide real-time results, making it suitable for intraoperative assessment and guiding surgeons during BCS.

In our research, we aimed to develop a novel and straightforward method for non-destructive and non-contact imaging of breast tissues. We initiated the process by illuminating the examined breast samples with a broad-spectrum halogen lamp, followed by scanning them using a HS camera. This allowed us to detect the spectral absorption bands of the studied BC samples and determine the optimal laser wavelength that induces fluorescence in these samples. To generate a unique fluorescence signature of BC areas, we employed LIF spectroscopy. We utilized a blue laser source to induce fluorescence with a higher yield in the BC samples. Simultaneously, the HS camera was employed to accurately measure the emission spectra of the examined specimens. The HS camera proved to be instrumental in extracting the optical features of the samples and capturing fluorescence signals. Its capability to perform spectrometry on distant target snapshots facilitated the acquisition of a series of images in cube format [40, 41]. These images provided valuable data on target brightness and irradiance within a confined spectral bandwidth of approximately 5 nm. Our approach enabled the identification of the optimal wavelength capable of discriminating between precancerous and cancerous tissues. The results obtained from our two-imaging setup, along with the scattering and emission spectra recorded by the HS camera, allowed for successful confirmation of tumor locations. Furthermore, we implemented rapid image segmentation using the k-mc approach with contour delineation (K = 8). The motivation behind utilizing the k-mc with delineation approach in our study lies in its potential to enhance the accuracy and efficiency of BC detection and delineation [42]. Traditional imaging techniques often face challenges in precisely identifying the boundaries of malignant tissue and distinguishing it from healthy tissue. This limitation hampers surgical interventions, as it becomes difficult to ensure complete tumor removal and minimize the risk of tumor-positive resection margins. By incorporating k-mc into our methodology, we aim to overcome these limitations. K-mc is an unsupervised machine learning algorithm that can effectively classify data into distinct clusters based on their similarities [43]. In the context of BC detection, this clustering approach enables the differentiation of cancerous and non-cancerous regions based on the fluorescence signals acquired from the examined breast samples. The application of k-means clustering in our delineation approach offers several advantages. Firstly, it provides an automated and objective means of segmenting the breast tissue into different clusters, thereby facilitating the identification of malignant regions. This assists in precise delineation and localization of tumors within the breast tissue. Additionally, the clustering approach can handle large datasets efficiently, enabling rapid processing and analysis of fluorescence signals, which is crucial in a clinical setting where time is of the essence.

Through our research, we have developed and presented a novel method that integrates LIF spectroscopy, HS imaging, and k-mc for the differentiation and delineation of malignant and non-malignant breast tissues. This innovative approach holds great promise in enhancing the diagnostic capabilities of BC detection, ultimately leading to more accurate and targeted interventions. Furthermore, the non-destructive and non-contact nature of our method offers distinct advantages in preserving the integrity of the examined tissue samples, allowing for further analysis and potential follow-up studies. By combining advanced imaging techniques and machine learning algorithms, our work contributes to the advancement of BC early detection and clinical practice.

## Materials and methods

Between October 2019 and February 2020, the investigation took place at the "Kobri El Koba Military Complex Hospital." The work received ethical approval from the Faculty of Medicine at Ain Shams University in Egypt and complied with the Declaration of Helsinki's Ethical Principles for Medical Research Involving Human Subjects. P.T.REC/009/003156 is the reference number. Before starting data collection, each respondent read and agreed to two copies of a written agreement. Thirty separate patients' normal tissue and breast tumor were removed for the study that was just presented. The samples under investigation are pathology slides that have BC diagnoses. Our study concerned females who had BC growth and underwent a full breast extermination procedure. Patients who met the following requirements were included in the study: (1) a recent diagnosis of breast cancer confirmed by needle biopsy; (2) no prior chemotherapy or hormone therapy; (3) tumor size between 1–3 cm; (4) unilateral BC; (5) no prior breast surgery; and (6) no motion artefacts. Both MRI and ultrasound were used by a radiologist to confirm the BC diagnosis. The patient information for this research is summarized in Table A 1 in the Appendix. Following the rigorous procedure, the patients were randomly selected, and breast tumor samples were obtained for histological evaluation. The tumors were prepared for HS imager from the removed breasts after breast concealment. Breast tumor HS images were collected. These experimental breast tissue samples were sliced and placed in an ice box with deionized saline with measurements of (200 mm × 300 mm) and sample thickness of 3 ~ 5 mm. This extracted biopsy consists of normal tissue and the tumor. Analysis was conducted at 25 °C, a standard sample temperature of 23 to 25 degrees Celsius predicted before each preparatory and kept in the fridge up to -70 °C. It was generally accepted that the region 50–100 mm away from the tumor was healthy, and pathology results supported this belief.

**Fig. 1:**
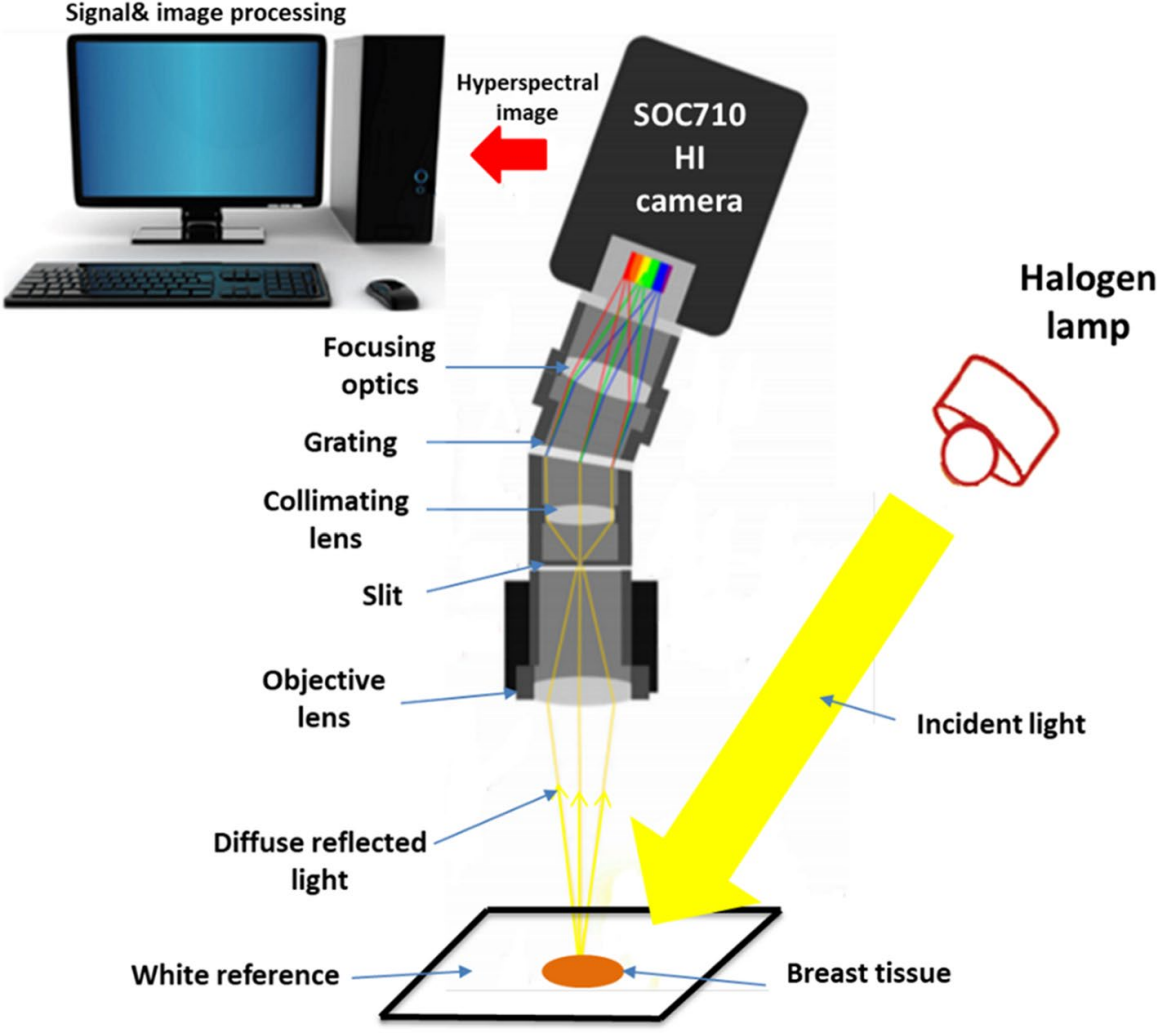
The HS optical imaging system schematic illustration for breast absorption characteristics computations

**Fig. 2:**
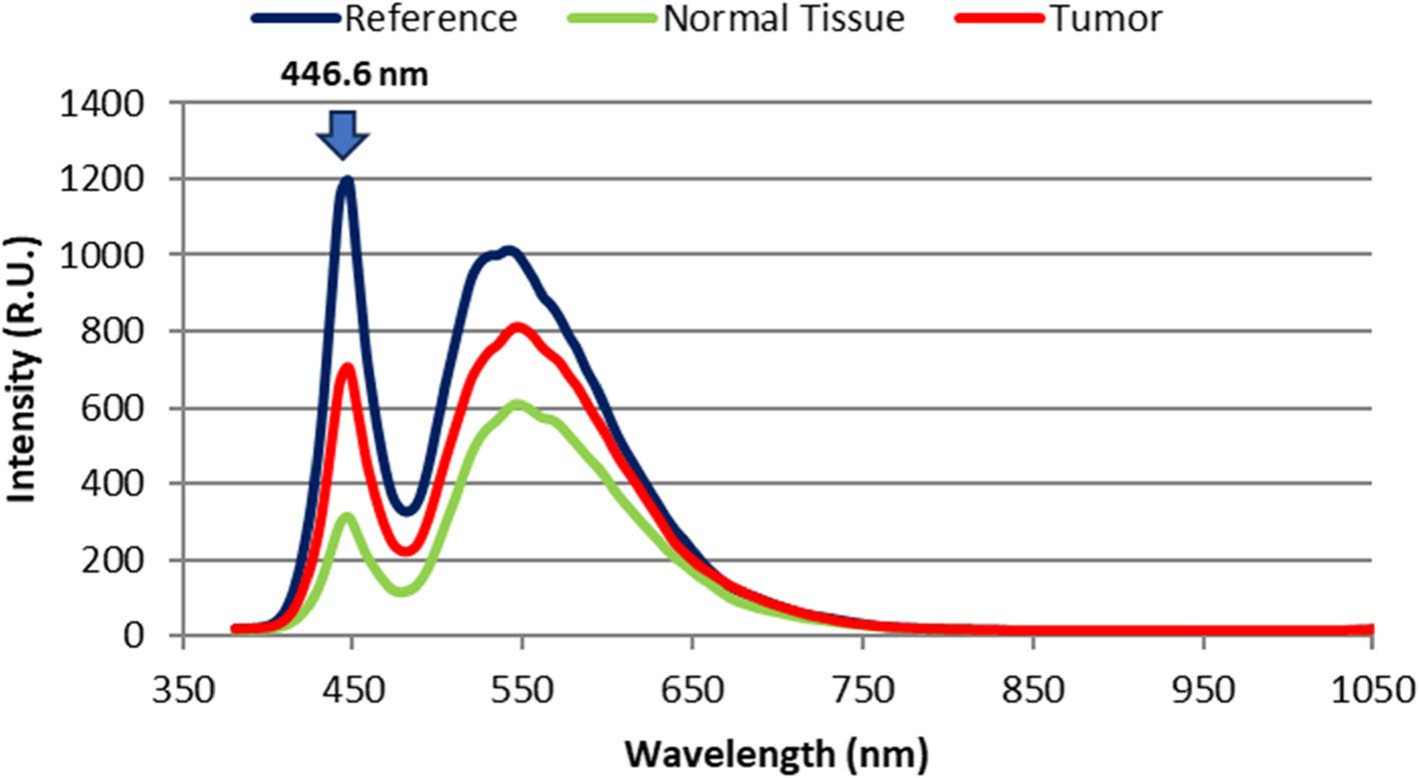
The combined average of the 10 breast test samples' diffused spectra characteristics

**Fig. 3:**
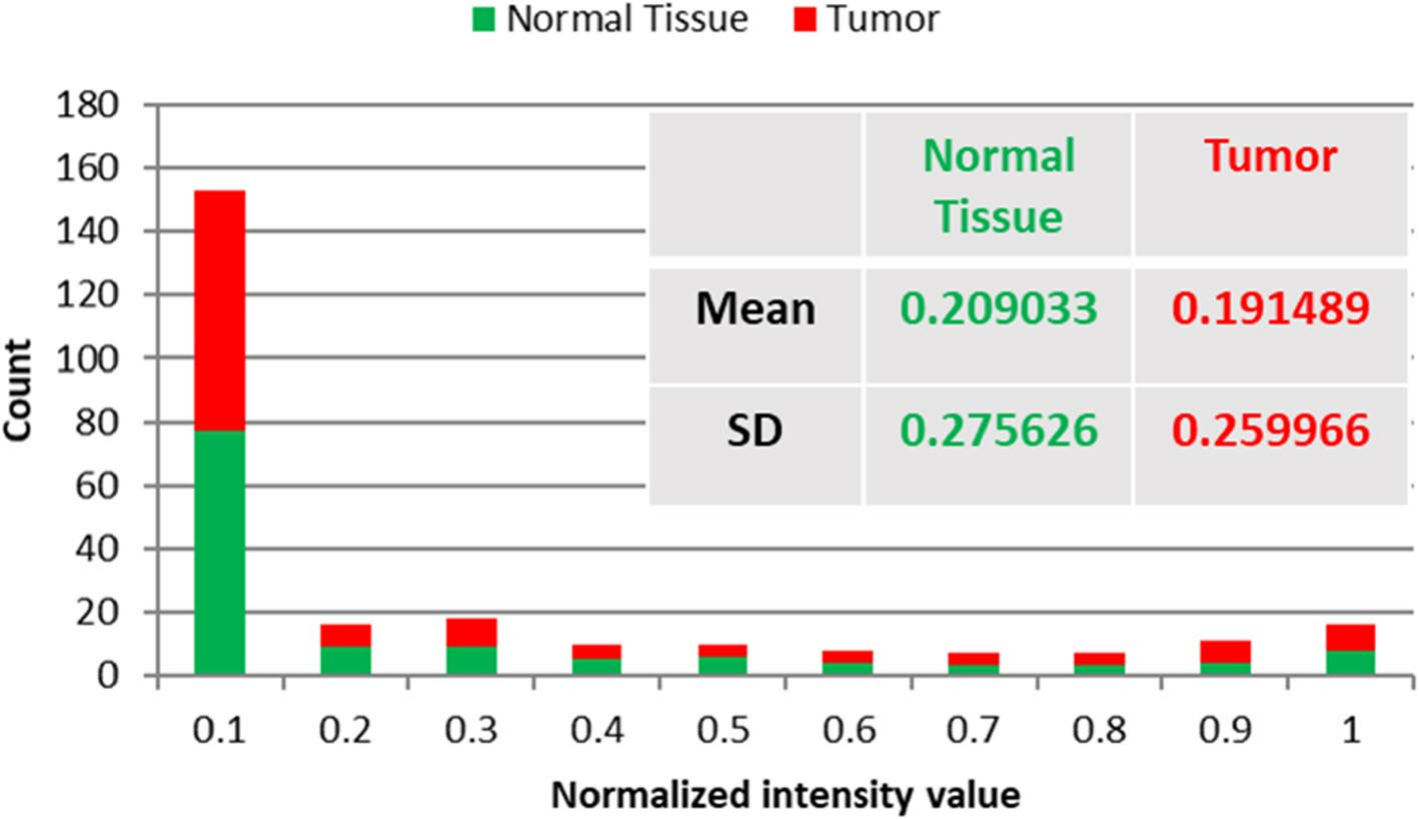
The average compound of the measured histogram, Mean and SD for the ten breast tested samples

Before using our proposed delineation technique, two distinct steps of laboratory research on breast cancer (BC) specimens were conducted. In the initial phase, we focused on determining the common absorption wavelengths of the 10 BC samples across the visible-near infrared (VIS/NIR) range. This was achieved through HS scanning and the utilization of broad-spectrum illumination at an acceptable level. Subsequently, in the second setup, we employed a laser beam with a wavelength falling within the absorption bandwidth identified in the first phase. This laser beam was used to excite the BC specimens, and HS imaging was employed to capture optical signature readings based on LIF at the same distance. The schematic representation of the HS first setup is shown in Fig. 1. We should calibrate the HS imagery to produce the highest S/R outcome before beginning our experimental work. To achieve geometric calibration, we first employed a ruler with known geometric properties. By rotating the utilized F/10 lens until we achieved optimal focusing, we were able to rectify the HS picture for any geometric aberrations. The background response is then determined using a spectral picture taken from a white reference sheet with a high reflectivity standard. A non-reflective dark cover is entirely fitted over the camera lens to generate the black appearance. The formula below is then used to calculate the relative reflectance for the captured images using these two acquired reference images [44].

1 $${\mathrm{I}}_{fc}=\frac{{\mathrm{I}}_{\mathrm{oc}}-{\mathrm{I}}_{\mathrm{Dc}}}{{\mathrm{I}}_{\mathrm{Bc}}{-\mathrm{I}}_{\mathrm{Dc}}}$$

where I_*fc*_ is the corrected spectra response captured image, I_*oc*_ is the raw spectra response captured image, I_*Dc*_ is the dark recorded image, and I_*Bc*_ is the white recorded image. The visible-NIR F/1.9 lens of the SOC710 HS line scan camera was initially focused on scanning the whole landscape for each investigated breast tissue. After the final line of scanning was finished, the references in black and white were recorded down. The HS system's wavelength dispersion part is just a grating with a CCD detection. The spectrograph divides the dispersed spectrum generated by the reflected beam into several directions while retaining its spatial information based on wavelength. After the dispersed light has been established onto the sensor the scanning line's spatial data (520 pixels per row) and spectral data are separated into separate dimensions. On the SOC710 imager in use, the sensor has been moved behind the lens. The result is a full 3D HS picture cube. Figure 2 displays the average diffused spectra characteristic profiles gathered by the SOC710 HS camera for the ten breast samples. On the other hand, Fig. 3 presents the statistical measures of our HS imaging setup, including histogram, mean, and standard deviation (SD), providing an overview of the tested samples.

**Fig. 4:**
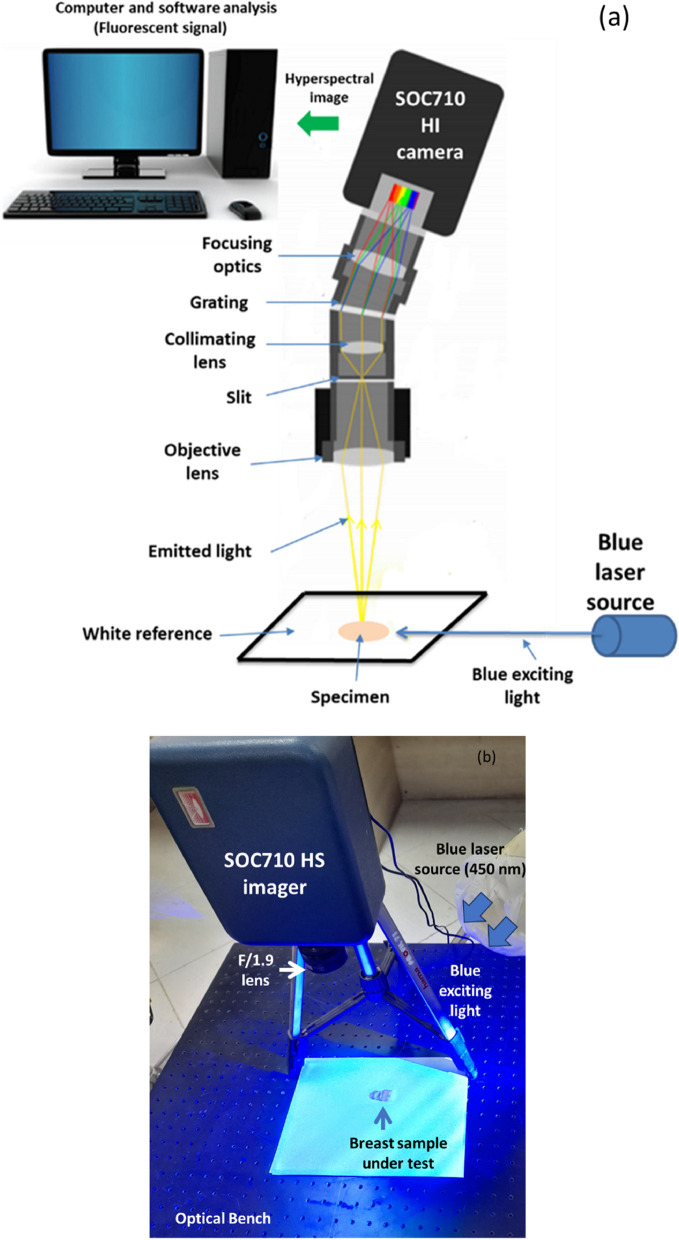
**a** HS optical imaging system schematic illustration based on LIF; **b** The exact configuration setup

**Fig. 5:**
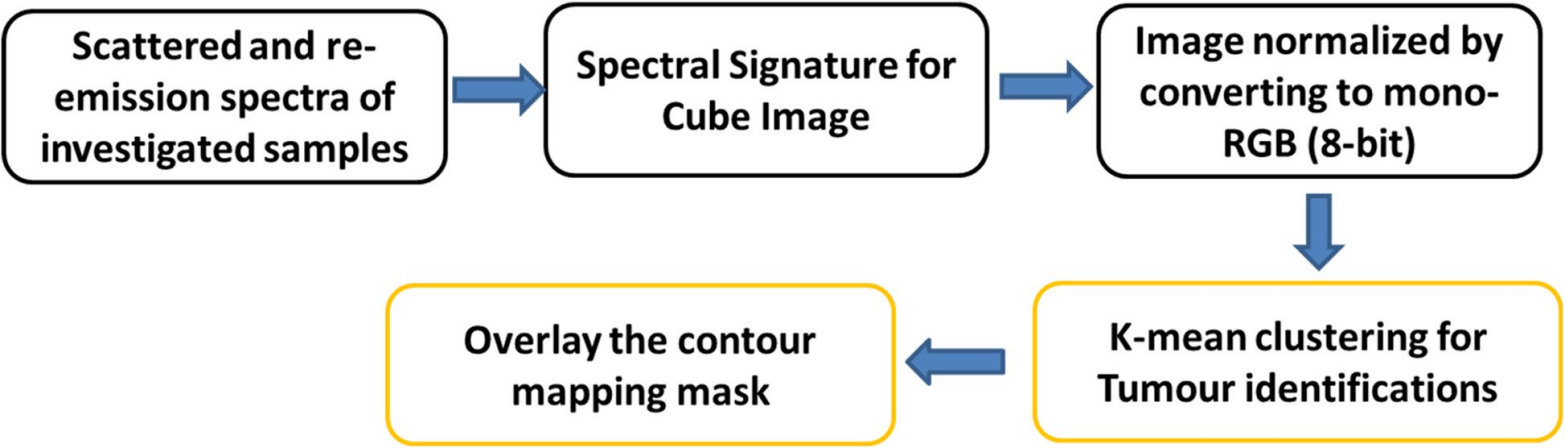
Image processing algorithm sequence and tool block diagram for this investigation utilizing the HS system for tumor detection based on LIF

Examining the resultant cube image and the related statistical analysis, as shown in Figs. 2 and 3, reveals that the examined BC samples exhibit considerable absorption in the spectral range between 440 and 460 nm. As a consequence of this finding information and the distribution outcomes of pixel intensities in the investigated images, we decided to continue with LIF testing using a laser source whose wavelength is 450 nm (blue in color), which is the mean of the characteristic absorption spectra of the BC samples under investigation. At 450 nm, we found that the difference in absorbance characteristics between the two types of tissues was larger compared to the difference observed at 560 nm. This larger difference in absorbance intensity at 450 nm facilitated the differentiation process between tumor and normal tissue. By choosing this wavelength for our LIF approach, we were able to excite a broader range of endogenous fluorophores present in the BC samples, resulting in a more distinct and discernible fluorescence signal compared to normal tissue. Moreover, Shorter wavelength stimulation has been found to excite more bands, which improves the ability to detect malignancies [25, 27]. At shorter wavelengths such as 450 nm, the energy of the incident light is higher, and it can interact with a broader range of fluorophores in the tissue [45, 46]. This includes a wider variety of endogenous molecules that are often associated with cellular activities and biochemical processes. As a result, shorter wavelength stimulation can excite more fluorescent bands in the tissue, leading to a more informative fluorescence signature. In the context of detecting malignancies, cancerous tissues often exhibit changes in cellular composition compared to normal tissues. These changes can result in differences in the abundance and distribution of certain fluorophores and chromophores, which can be detected through their unique fluorescence response to shorter wavelength stimulation. The suggested LIF imaging mechanism consists of a HS camera (Surface Optics, SOC710, USA) with a VIS/NIR wavelength range (380–1050 nm) and a 50-mW commercial blue laser (450 nm) with a 1.1 mm beam size and 0.6 m rad beam divergence. The blue laser source used to excite the biopsy samples was roughly 100 cm away from the optical bench and about 90 degrees off-axis from the HS camera. The camera's installed lens is (Schneider, 400–1000 nm, Germany). The schematic representation of the HS setup with LIF with the exact imaging configuration is shown in Fig. 4 (a) and (b), respectively. Each gathered cube image had 128 spectral frames with a spatial resolution of fewer than 40 microns and a spectral resolution of 5 nm. As a consequence, the system was lighted, and all elements were fixed throughout all of the study trials. The utilized optical lens had a viewing field of 10°, capturing a picture with dimensions of 6 cm × 8 cm at 50 cm, which is appropriate for high focusing for the HS camera and the analyzed samples. Figure 5 depicts the flowchart image processing path that led to our encouraging results, analysis, and ultimate conclusion. A device (laptop) that runs software (HS-Analysis TM Data Analysis) managed the linear scanner's motors, adjusted exposure, and gathered the emission characteristics data extracting the LIF information for different samples of the biopsied specimens on the white reference that settled all on the optical board.

After implementing image acquisition, we adopted an approach known as 8-bit normalization to scale the intensity values of each pixel to an 8-bit range (0–255), which is equivalent to a typical RGB picture. This normalization technique serves multiple purposes, including facilitating data visualization and analysis, as well as ensuring compatibility with our processing tool, DADiSP 6.5. By applying this step, we effectively reduced the influence of variability and noise in the gathered data, while ensuring equal weighting of all spectral bands in the subsequent clustering analysis. In the image processing proposed work, we used the K-mc intention is to establish k centers, one for every grouping. Typically, they should be placed as widely apart as feasible in order to improve recognition rate and limit the occurrence of false signals. Now next procedure is to associate every point within a particular set of data with the nearest cluster center. In this approach, each category will have one center point, and the outcome gets progressively consistent as iterations rise, indicating that they have reached an assent. According to our histogram computations for the examined breast specimens, we determined the optimum choice of k = 8 for the k-mc applied to the normalized HS image data. By selecting this value, we aimed to strike a balance between accuracy and computational efficiency. Consequently, our application of k-mc to the normalized HS image data with k = 8 allowed us to effectively analyze and classify the breast specimens in an efficient and accurate manner. The fundamental steps in the K-mc (K = 8) algorithm for selecting clusters are described in Eqs. (2) and (3) [47, 48]. The optimal solution, J^*^(*V*), is first minimized by this approach utilizing:

2 $${J}^{*}(V)=\sum\nolimits_{i=1}^{m}\sum\nolimits_{j=1}^{{m}_{i}}{\left(\Vert {z}_{i}-{ce}_{j}\Vert \right)}^{2}$$

where *“||z*_*i*_* – ce*_*i*_*||*” is the Euclidean distance between *z*_*i*_ and *ce*_*i*_, “*m*” is the number of cluster centers, “*m*_*i*_” is the number of data points in *i*^th^ clustering, *Z*_*i*_ is the collection of data sets to be clustered, and *ce*_*j*_*.*

Is the set of d-dimensional centroids. The formula for the minimum-distance classifier is used to generate the new cluster centroid, *ce*_*i*_ [47]

3 $${ce}_{j}=\frac{1}{{m}_{i}}{\sum }_{j=1}^{{m}_{i}}{z}_{i}$$

The DADiSP 6.5 software served as the primary foundation for the image processing algorithm sequence (DSP Development Corporation, USA). With the use of our method for processing images, accurate image spectral data was made available that could be utilized to cluster the tumor from the surrounding healthy tissue using the LIF method.

## Results and analysis

**Fig. 6:**
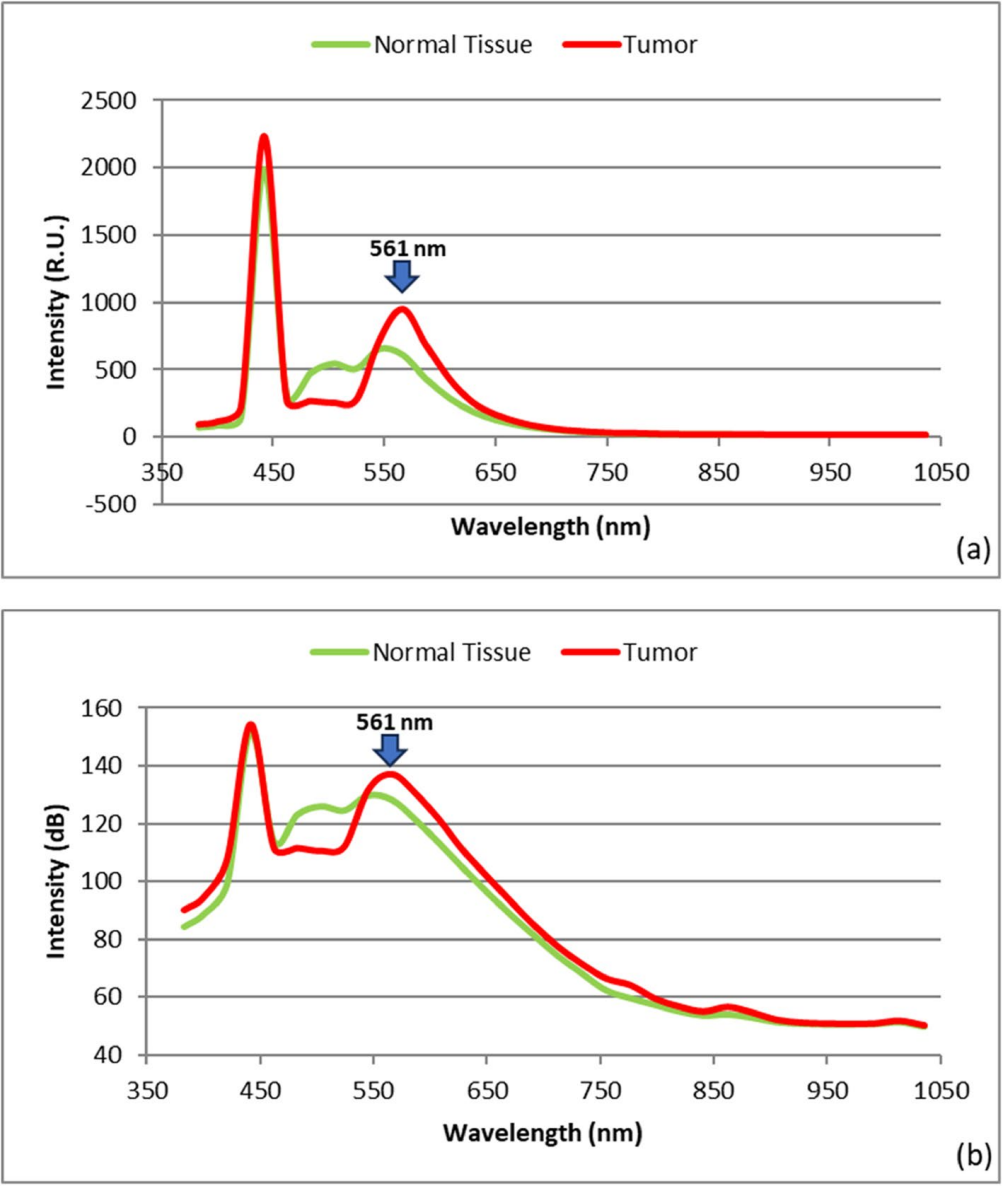
Average LIF signature for the BC tissue samples; **a** normal and tumor overlaid; **b** Spectrum characteristic scaled logarithmically

**Fig. 7:**
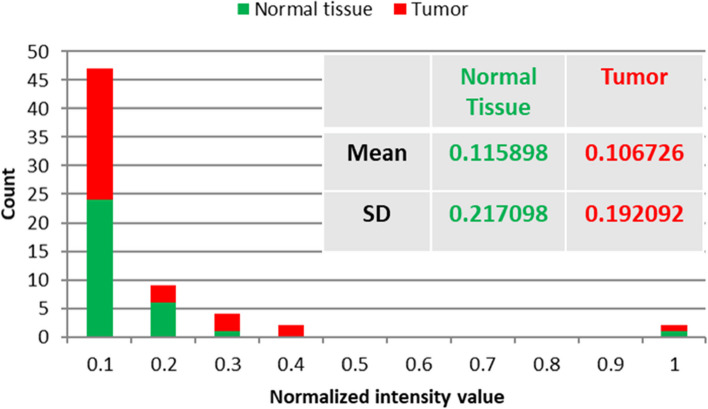
Visual representation of the distribution of fluorescence intensity values across the 10 tested samples, including histogram computation and related mean and SD

The proposed LIF setup for BC detection involves the response of specimens to 450 nm source excitation light. The HS camera computes the emitted spectra of light, allowing for the selection of the appropriate wavelength for tumor classification using our algorithm. Fluorescence occurs when molecules are excited by a steady light source, and the difference in fluorescence intensity between normal and malignant breast tissues could be our optical marker for our BC differentiation process. By utilizing our LIF approach with a wavelength of 450 nm, we induced a significantly greater state in the investigated breast samples. The emission of the fluorescence signature allows the stimulated matter to return to its baseline state, and the emitted photons represent all potential energy transitions of the stimulated material. The SOC710 HS imager accurately measures the LIF signature, which can be utilized to identify the biochemistry of tissue structure. The fluorescent data was collected and managed using the HS-Analysis TM Data Analysis software. In our HS imaging investigation, we analyzed a total of 10 BC specimens, collecting HS images from each patient. A quantitative analysis comparing the emitted spectra of normal and cancerous tissue was conducted based on the emitted radiation of the spectrum images. When the tested samples were illuminated with a blue laser source, they exhibited a high photoluminescence signature at longer wavelengths, as represented in Figs. 6 and 7 illustrates the statistical measures of our proposed LIF imaging setup for the tested samples, including the histogram, mean, and SD. The error bars in the figure represent the variation observed in the data.

**Fig. 8:**
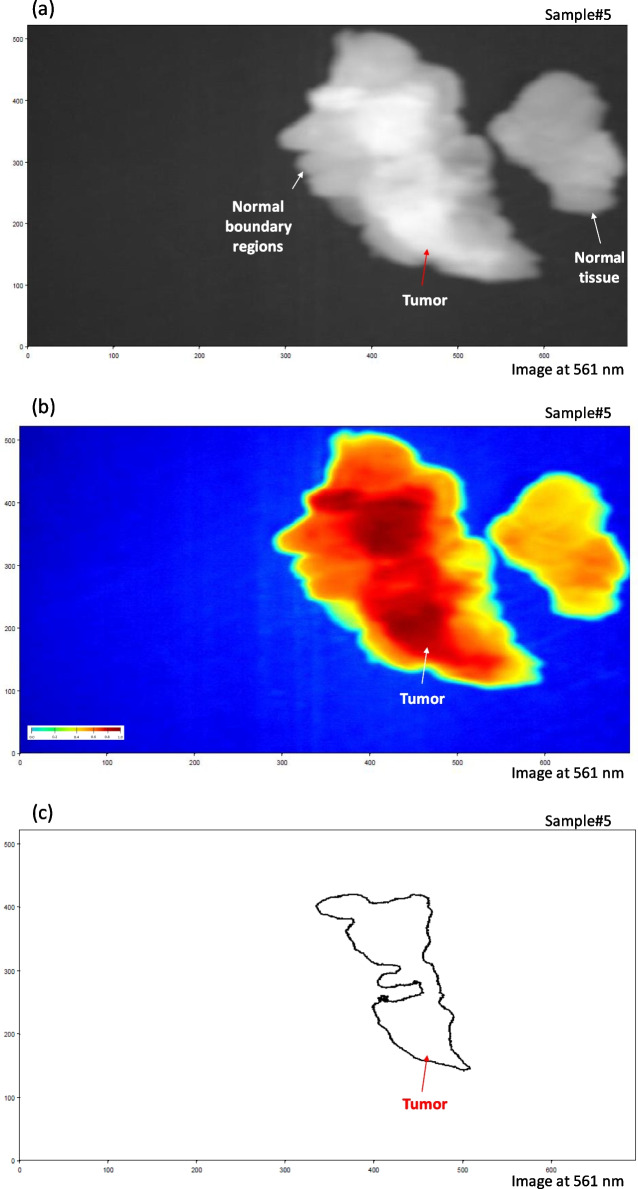
**a** The picture taken at 561 nm by the HS imager for sample #5; **b** The 561 nm image after applying image pre-processing; **c** The prepared Image with the K-mc (K = 8) on the given threshold value ≥ 1.7 for delineating the BC spots using our LIF results

Figure 6 demonstrates the fluorescence signature obtained when the sample was stimulated with a 50-mW blue laser source. It is observed that the fluorescence signature varies depending on the tested tissue sample. We identified a distinct wavelength, 561 nm, at which the maximum variation in emitted fluorescent signal occurs for both tumor and normal tissue. The average intensity value of the tumor at 561 nm is approximately 136 (dB), while it is about 127 (dB) for the normal tissue. This fluorescence signature at 561 nm serves as a crucial guide for selecting the optimal spectral image that can effectively differentiate between the tumor and normal tissue, thereby enabling accurate optical diagnosis of BC tissue. Figure 7 illustrates the calculation of histogram, SD, and mean, which provide valuable insights into the range and distribution of fluorescence intensities. These statistical measures can offer information about tissue properties and the presence of specific biomarkers associated with BC. Our analysis revealed that the mean and SD values for tumor tissue are 0.106726 and 0. 192,092, respectively, while for normal tissue, the corresponding values are 0.115898 and 0.217098. These findings contribute to a better understanding of the differences in fluorescence characteristics between tumor and normal tissue, aiding in the development of more accurate BC detection method using our proposed LIF approach.According to our imaging results, we selected this optimal fluorescence emission frequency for implementing our image processing methodology. This involved utilizing image segmentation with K-mc to analyze the variance in emitted spectra in response to the spectral image corresponding to the fluorescence signature at 561 nm for breast tissue sample #5 (Fig. 8(a)). In order to facilitate the image processing of the studied specimens, a pre-processing approach was applied to the 561 nm spectral image, as depicted in Fig. 8 (b). The application of calculated contour mapping of the spectral image (561 nm) with K-mc (K = 8) is clearly illustrated in Fig. 8 (c), demonstrating the differentiation and characterization of normal tissue using the 0.561 µm spectral image.

**Fig. 9:**
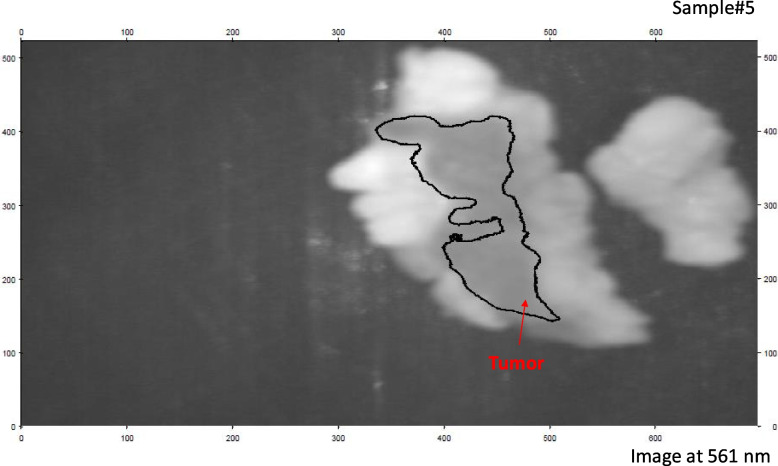
Applying a contour mapping mask for the BC regions based on the specified threshold to the taken image for sample #5 at 561 nm based on LIF outcomes

Figure 8 illustrates our successful detection of the malignant area and its clustering relative to the normal tissue, utilizing the emitted signal intensity difference at 561 nm. By applying our classification method based on fluorescence, the tumor location induced by the blue laser was clearly discernible. Additionally, Fig. 9 demonstrates the application of a contour mapping mask to identify the BC regions based on a specified threshold applied to the 561 nm image captured by the SOC710 HS camera on samples #5.

**Fig. 10:**
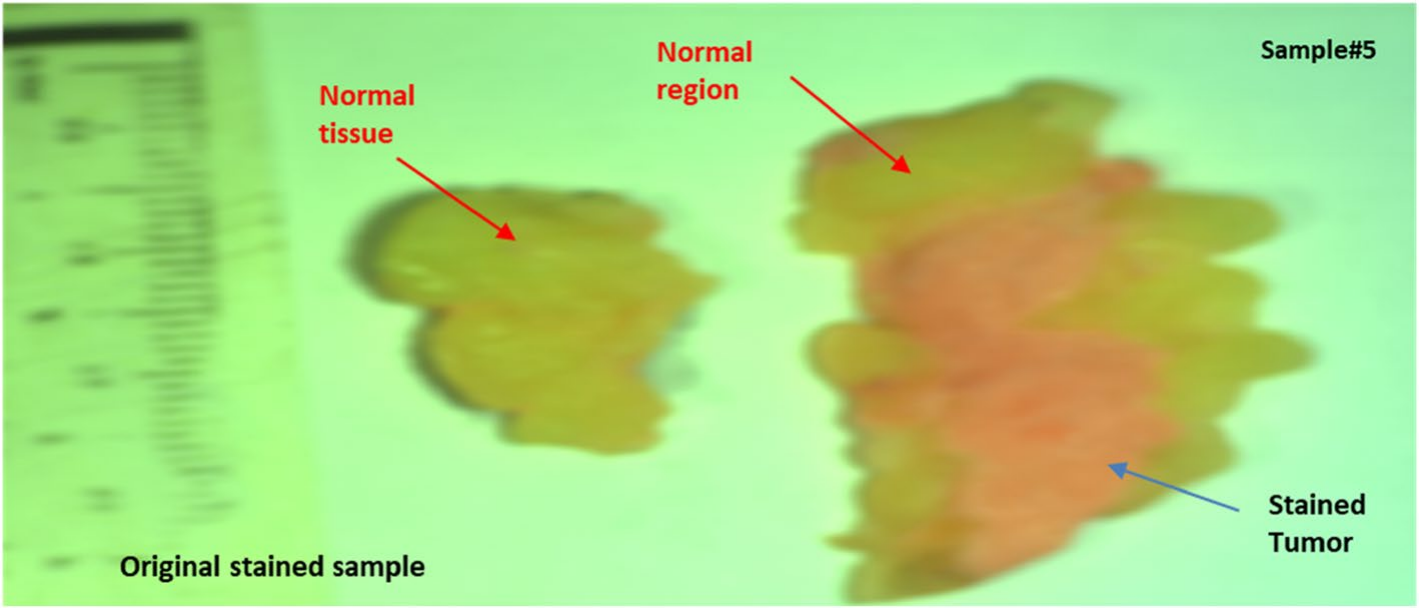
Ordinary camera's RGB pictures of an original stained sample #5 after the pathology examination

As shown in Fig. 9, we were able to overlay a contour mapping mask over the investigated sample #5 based on the specified threshold for the immediate grouping of malignant breast tissues.After the pathology examination, the effectiveness of the proposed LIF system technique was evaluated by comparing the results with the findings from the pathologist. The RGB image for breast sample #5, following the pathology analysis, is presented in Fig. 10.

Comparing our results shown in Fig. 9 with the pathology examination displayed in Fig. 10, we might apply our methodology using LIF to unknown breast samples with promising outcomes in BC instant detection. BC diagnosis demands utmost sensitivity and specificity, making the binary classification approach a vital aspect of our study [42, 49]. Our meticulous examination at the wavelength of 561 nm has yielded high-quality outcomes. By comparing these results with the gold standard histology examinations, we calculated True Positive (TP), True Negative (TN), False Positive (FP), and False Negative (FN) values to assess pixel accuracy. Any misclassification of a malignant pixel in the histology map was labeled as FN, while any erroneous identification of non-cancerous tissue as cancerous was recorded as FP. These evaluations were performed individually for each of the ten breast samples. our method demonstrated remarkable performance, with an average sensitivity of 94.33% and an average specificity of 97.14% across all ten breast samples. These results indicate a highly reliable and accurate BC detection capability, showcasing the effectiveness of LIF with HS Imaging and k-mc approach. Table 1 summarizes the sensitivity and specificity values for each sample:

**Table 1:** Sensitivity and Specificity of the proposed LIF classification for the ten samples at 561 nm

Sample	Sensitivity (%)	Specificity (%)	FN ratio (%)	FP ratio (%)
1	96.2	97	3.8	3
2	93.4	95.8	6.6	4.2
3	95	98.1	5	1.9
4	93	97.6	7	2.4
5	94.6	98.5	5.4	1.5
6	93.3	97.7	6.7	2.3
7	95.2	97.2	4.8	2.8
8	94.5	96.4	5.5	3.6
9	93.6	97.5	6.4	2.5
10	94.5	95.6	5.5	4.4

**Fig. 11:**
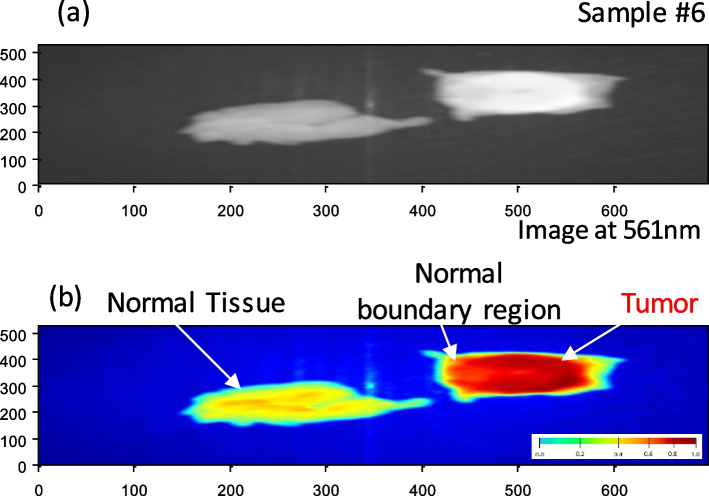
**a** The picture recorded at 561 nm by the HS imager for sample #7; **b** The 561 nm image after applying image pre-processing for delineating the BC spots using our LIF results

**Fig. 12:**
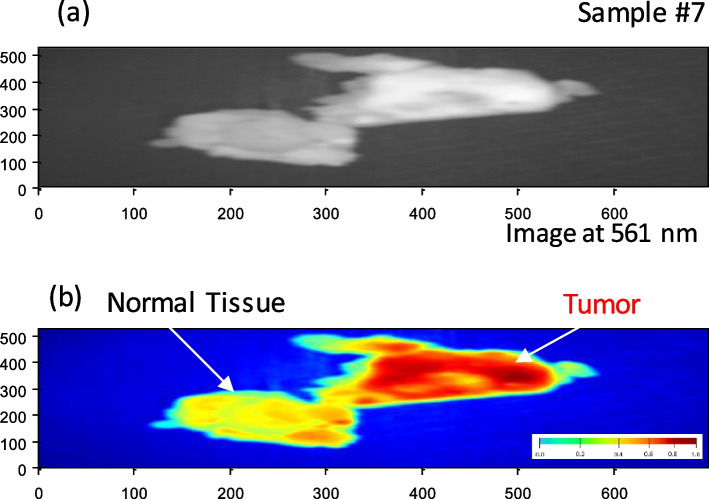
**a** The picture taken at 561 nm by the HS imager for sample #7; **b** The 561 nm image after applying image pre-processing for delineating the BC spots using our LIF results

**Fig. 13:**
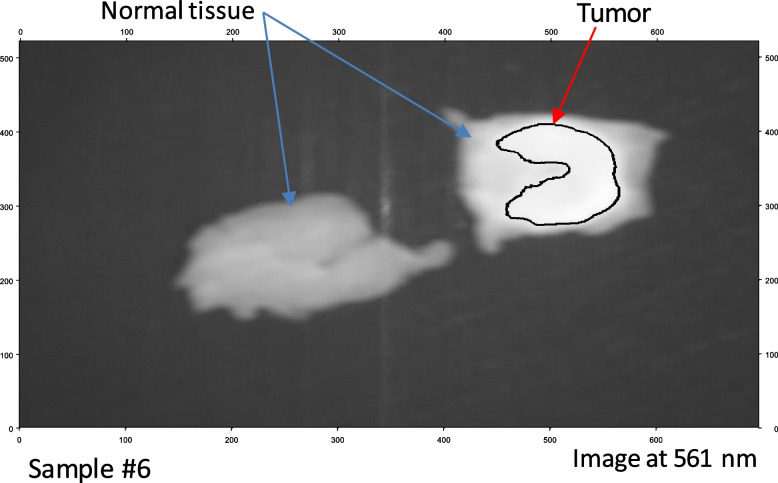
Applying the proposed k-mc and a contour mapping mask for the BC regions based on the specified threshold to the taken image for sample #6 at 561 nm based on LIF outcomes

**Fig. 14:**
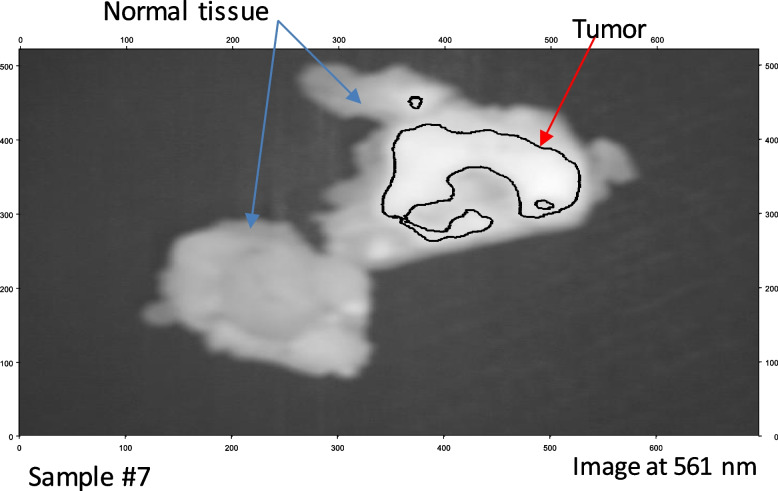
Applying the proposed k-mc and a contour mapping mask for the BC regions based on the specified threshold to the taken image for sample #7 at 561 nm based on LIF outcomes

According to Table 1, These promising outcomes indicate that the photoluminescence signature effect after stimulation by the blue light laser enables novel label-free fluorescence BC detection and delineation. Each material clearly exhibits a unique photoluminescence signature. We may use these characteristics to instantly detect the tumor's location. The fluorescent signal intensity may assist considerably in distinguishing between the tumor and the surrounding cells at this candidate fluorescence frequency (561 nm). Finally, we applied our LIF approach to BC samples 6 and 7, which confirmed our proposed technique. We applied image preprocessing to the selected image related to the wavelength mentioned in the cube image related to these samples. Figures 11 (a) and 12 (a) show the selective 561 nm spectral image and the demarcated normal tissue from BC after applying a pre-processing approach to improve image size and quality, as shown in Figs. 11 (b) and 12 (b). Figures 13 and 14 show the application of a contour mapping mask for the BC regions based on the specified threshold after applying the proposed K-mc (K = 8) approach to the 561 nm image taken by the SOC710 HS camera on samples 6 and 7, respectively.

We successfully characterized and clustered, as shown in Figs. 11 and 12, BC with respect to the normal tissue for samples 6 and 7 using our fluorescent stimulation experimental setup by a blue light laser source. As demonstrated in Figs. 13 and 14, we applied our imaging approach based on the K-mc technique to the 561 nm emitted spectral image, which is selected according to our results to be a marker to cluster between the tumor and normal tissue. Finally, we could overlay a contour mapping mask based on the given threshold for the instant grouping of non-cancerous breast tissues on the studied samples 6 and 7, respectively. According to our pilot study, we have developed a novel approach that relies on LIF with HS imaging and k-mc. Our proposed method has provided promising results in distinguishing between cancerous and healthy breast tissues without the need to use exogenous dyes. Using our proposed LIF spectroscopy, we have been able to detect and analyze the fluorescence emitted from tissues in response to laser excitation. By carefully selecting the excitation wavelength (450 nm) and employing a hyperspectral imager, we collected detailed fluorescence data across a range of wavelengths, capturing valuable information about BC diagnosis. One of the significant advantages of our approach is that it eliminates the need to use exogenous dyes for tissue staining. While some previous studies have employed dyes like Rhodamine 6G, an activatable polymeric or sodium fluorescein as presented in [25, 27, 32], our method utilizes endogenous fluorophores present within the tissue itself. This not only avoids the potential toxicity and hazards associated with exogenous dyes but also simplifies the diagnostic process by making it non-invasive and reducing the preparation steps which includes fluorescence detection equipment and spectral analysis software. Moreover, the use of k-mc in our methodology further enhances the accuracy of tissue segmentation and BC detection. By automating the segmentation process, k-mc streamlines the analysis and ensures our objective results [50–52]. The advantages of our pilot study for BC using label-free fluorescence, which leverages LIF with HS Imaging and K-mc, can be summarized as follows:

- ➢ Non-invasive and label-free: Our method utilizes endogenous fluorophores within the tissue, eliminating the need for exogenous dyes and making the diagnostic process non-invasive and label-free.
- ➢ Reduced risk and hazards: By avoiding the use of exogenous dyes, our approach mitigates potential risks and hazards associated with dye administration, ensuring patient safety and comfort.
- ➢ Objective results: The combination of LIF and k-mc offers objective results, enhancing the reliability and consistency of tissue segmentation and BC detection.
- ➢ Potential for real-time applications: The non-invasive nature of our proposed method and the rapid data processing provided by K-mc open the possibility for real-time intraoperative applications, enabling prompt decisions during BC surgical interventions.

By avoiding the limitations associated with exogenous dyes, our method offers a safer, more efficient, and accurate alternative for early BC diagnosis, which could aid clinical pathological inspectors in making an instant estimation.

## Discussion

Clinically pathological analysis has recently greatly benefited from accurate disease diagnosis as a method to assess the pathological alterations in cells. The current usual protocol is biopsy [53]. However, one drawback of a biopsy is that a sample of the body's tissue is taken out. Biopsy causes a great deal of physically agony to sufferers even though it is one way to diagnose diseases. In some severe forms, this procedure may also take a long time and result in inaccurate or delayed final diagnosis. The patients' desire to lower the cost of medical care is another reason to minimize the requirement for the surgical setting that needed extracting tissue samples and doing histology. So, prompt diagnostic information is required in order to reduce the amount of time the patient has wait for a response and, at the same time, to lessen any potential spiritual harm that might happen [54]. In the recent times, attention has turned to an innovative approach of disease detection called "in-vivo" description of disease using optical technology. With accuracy, responsiveness, and selectivity, the LIF approach can be automated and provide real-time detection and discrimination. As a non-invasive monitoring technique that can locate and identify abnormal tissue areas in real time, LIF have a significant impact on the identification of malignancies [55]. Spectra typically have a higher predictive precision than a judgment made only on the basis of a biopsy. Repeated biopsies may not be necessary as often thanks to LIF emission spectra [56]. Fluorescence intensity can indeed serve as a differentiating factor between malignant and normal tissue in our proposed studies, indicating the presence of specific molecules, metabolic activities, and structural changes within the tissue [57–59]. In the context of BC, alterations in the concentration and distribution of fluorophores, molecular composition, cellular metabolism, and tissue architecture are often observed in malignant tissues compared to normal tissues. These differences can result in changes in the quantum yield of fluorophores present in the malignant tissue. By quantifying and analyzing the differences in fluorescent intensity, we can propose an approach for BC detection and discrimination. This allows us to explore the potential of fluorescence intensity as a valuable tool in identifying and characterizing BC.

**Fig. 15:**
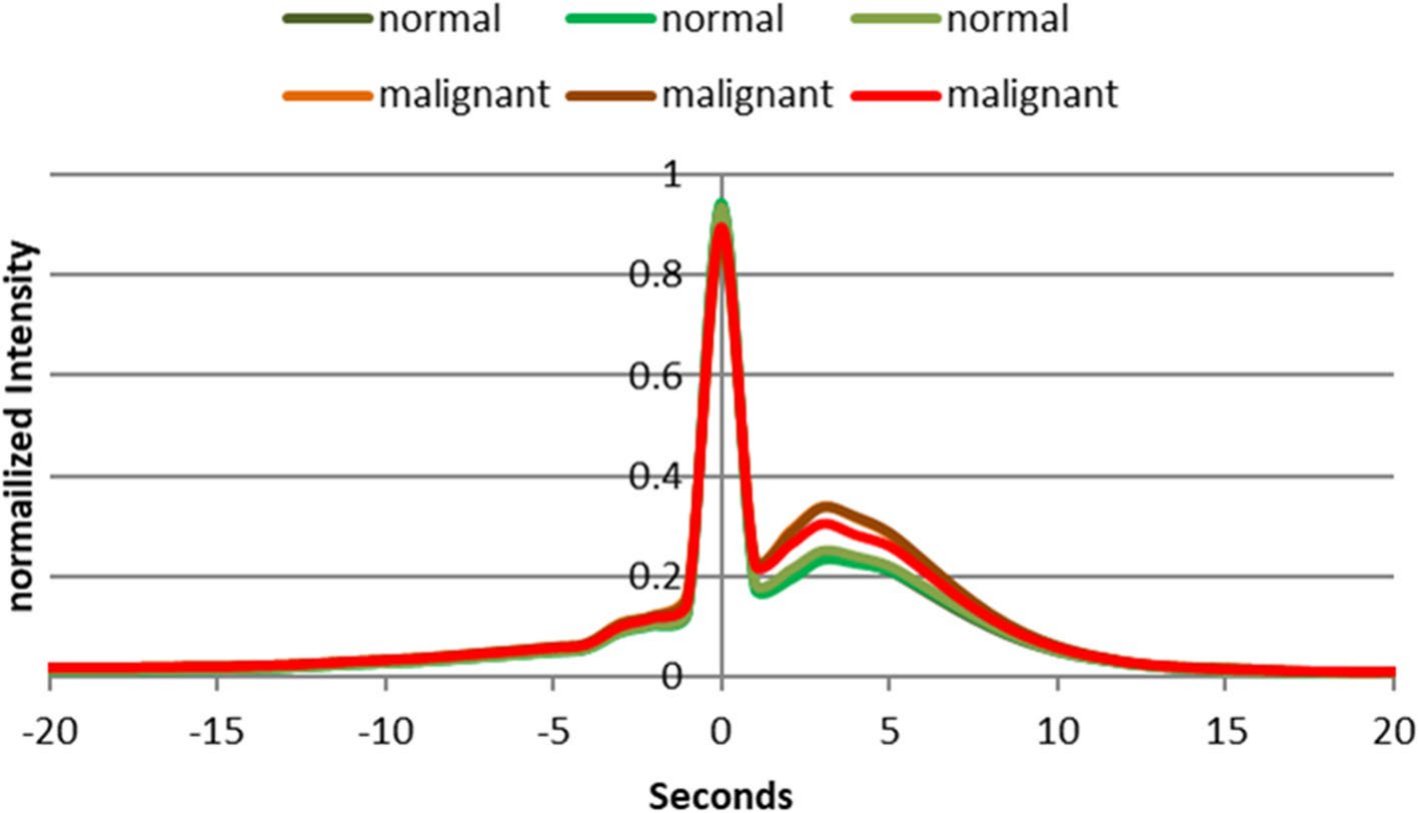
The fluorescent life time change between malignant and normal tissue with respect to the reference excited signal using the cross-correlation

We assessed our promising outcomes by calculating the fluorescence lifetime between the normal and malignant breast tissues. This involves recording the fluorescence intensity as a function of time after excitation. The average duration a fluorophore spends in the excited state before reverting to the ground state by producing a fluorescence photon is referred to as the fluorescence lifetime [60, 61]. By measuring the fluorescence decay curves for the two different molecular structures of both normal and malignant specimens for samples (5, 6, and 7), we could compute the cross-correlation between the normal and malignant fluorescence decay curves and measure the similarity between two signals as a function of the time delay between them, as shown in Fig. 15. This figure illustrates the time delay variance between the tumor and the normal breast regions, which evaluates our findings about the emittance intensity difference for the malignant tissue, which is presented as our optical marker for BC detection based on the proposed LIF approach.

Using the SOC710 HS imager enables us in our proposed imaging setup to get the fluorophore's chemical environment and elemental compositions both influence the Stokes shift's amplitude after stimulated the BC specimens by the blue (450 nm) light laser. The desired outcomes are achieved by applying an associated image processing algorithm using K-mc (K = 8) with contour delineation in the (380-1050 nm) spectral band. Our imaging processing grouping technique might be applied to cluster the tumor location in “in-vivo” operations during surgery with the LIF technique.

## Conclusion

Our study introduces an integrated technique for the classification of emitted hyperspectral (HS) images, aiming to detect cancerous tissue in a more efficient and less invasive manner. Traditional diagnostic methods, such as postoperative pathological approaches, have limitations in terms of complexity and time-consuming tissue preparation. In our approach, we first determined the common absorption wavelengths of the studied breast cancer (BC) samples using HS imaging and broad-spectrum illumination. Notably, we observed a significant absorbance difference in the range of 440 to 460 nm. Subsequently, we developed a customized laser-induced fluorescence (LIF) method utilizing blue laser excitation to measure fluorescence in both healthy and cancerous ex vivo human breast tissues. Our system achieved high classification outcomes, which were confirmed by pathology results, validating its effectiveness in detecting cancerous tissue. The combination of the HS classification technique and our research methodology enabled precise localization of tumors and the identification of structural changes within tissues. We found that extracellular protein emissions contributed significantly to the fluorescence spectra and that variations in their intensity could be utilized to detect tissue abnormalities. The use of a 450 nm blue laser source provided optimal observation of these differences. In addition to utilizing photoluminescence signature for BC detection, we also identified that signal intensity can serve as a distinguishing factor. Our findings showed that the intensity of malignant tissue is higher than that of normal tissue, providing an optical diagnostic marker for BC. This prospective noninvasive technique, made possible by HS imaging, allows for the examination and evaluation of a substantial portion of tissue without the need for invasive procedures or tissue sample removal. Furthermore, this technique has the potential to enhance a surgeon's visual capabilities and can be considered as a virtual biopsy tool, allowing real-time examination of suspected cancerous tissue during surgery. By combining our unique imaging methodology based on LIF spectrophotometric analysis with our K-mc strategy and contour delineation, we have laid the foundation for a new and efficient "in-vivo" description of diseases using optical technology, particularly in the early detection of tumors. This approach holds promise for improving diagnostic accuracy and guiding surgical interventions with enhanced precision.

### Supplementary Information

**Additional file 1: Table A1.** The Features Table for BC Investigated Samples Used in This Study's Experimental Approach.

## Data Availability

The authors stated and declare that all the datasets used and/or analyzed during the current study are available from the corresponding author on reasonable request to preserve the copyright. The authors stated and declare that all code exists and is available.
