# Supplementary material for: Delineation and detection of breast cancer using novel label-free fluorescence
Source: BMC Med Imaging. 2023 Sep 16;23:132. doi: 10.1186/s12880-023-01095-2 (PMC10505331; doi:10.1186/s12880-023-01095-2)
Supplement: Supplementary file 1 — Additional file 1: Table A1. The Features Table for BC Investigated Samples Used in This Study's Experimental Approach. [file 12880_2023_1095_MOESM1_ESM.docx]

**Appendix**

Table A1: The Features Table for BC Investigated Samples Used in This Study's Experimental Approach

| Patient No. | Lab. No. | BC type* | Age | Size of sample (cm) | Size of tumor (cm) | Tumor Grade | Tumor histology kind |
| --- | --- | --- | --- | --- | --- | --- | --- |
| Pat.#1001 | 154543 | C | 56 | 8 x 9 x 3.5 | 2.5 × 4 × 1.5 | I | IDC |
| Pat.#1002 | 152567 | D | 69 | 8.5 x 10 x 5 | 3 × 4 × 2.5 | II | IDC |
| Pat.#1003 | 407153 | C | 49 | 15 x 17x 3 | 1 × 1.3 × 1 | III | IDC |
| Pat.#1004 | ZK4900-22 | C | 60 | 16 x 24 x 9 | 7.5×14 | II | IDC |
| Pat.#1005 | ZK5435-22 | D | 94 | 10 × 14 ×8.5 | 2.5 × 2.5 & 0.6 × 0.5 & 1.5 × 1 | II | Multifocal IDC |
| Pat.#1006 | 152513 | B | 69 | 6 × 10.5 × 4.5 | 2.5 × 4 ×1.5 | I | IDC |
| Pat.#1007 | 162568 | D | 49 | 8.5 × 9.5 × 5 | 3 × 4 × 2.5 | II | IDC |
| Pat.#1008 | 467151 | B | 54 | 15 × 12 ×3 | 1 × 1.3 × 1 | III | IDC |
| Pat.#1009 | 153511 | B | 50 | 16 × 24 × 9 | 7.5 x 14 | II | IDC |
| Pat.#1010 | 182578 | D | 46 | 15 × 17 ×3 | 1 × 1.3 × 1 | III | IDC |
| Pat.#1011 | 567152 | B | 51 | 11 × 16 ×6 | 5.5 × 6 | II | IDC |
| Pat.#1012 | ZK4611-12 | B | 53 | 9 × 12 ×4.5 | 3.5× 2.5 × 3.5 | II | IDC |
| Pat.#1013 | ZK3635-32 | C | 56 | 8.5 × 11 ×5 | 3.5 × 4 × 2.5 | III | IDC |
| Pat.#1014 | 132520 | D | 58 | 13 × 18 × 3.5 | 1.5 × 2.3 × 2 | I | IDC |
| Pat.#1015 | 112567 | C | 60 | 14.5 × 21 × 9 | 3.5 × 2.5 × 4.5 | II | IDC |
| Pat.#1016 | 367132 | C | 55 | 12 × 21 × 6.5 | 5.5 × 6.5 | II | IDC |
| Pat.#1017 | 123611 | C | 59 | 9 × 18 ×3.5 | 5.5 × 2.5 × 1.5 | II | IDC |
| Pat.#1018 | 192471 | C | 72 | 8.5 × 12 ×5 | 2.5 × 4 ×2.5 | II | IDC |
| Pat.#1019 | 267132 | C | 69 | 13 × 17 × 3.5 | 1.5 × 2.3 × 2 | II | IDC |
| Pat.#1020 | 167511 | C | 67 | 11 × 16 × 9.5 | 3.5 × 2.5 × 4.5 | II | IDC |
| Pat.#1021 | 167571 | B | 59 | 6 × 9.5 × 3.5 | 2.5 × 3.5 × 2.5 | I | IDC |
| Pat.#1022 | 257111 | D | 45 | 6.5 × 3.5 ×6.5 | 3 × 4.5 × 2.5 | II | IDC |
| Pat.#1023 | ZK6611-01 | B | 52 | 11 × 5.5× 11 | 1 × 3.5 × 2.5 | III | IDC |
| Pat.#1024 | ZK3985-02 | B | 53 | 14 ×20 × 7.5 | 5.5 × 11 | II | IDC |
| Pat.#1025 | 122541 | D | 49 | 14 ×18 × 3.5 | 2.5 × 6.5 × 2.5 | III | IDC |
| Pat.#1026 | 112432 | B | 55 | 12 × 19 ×6.5 | 5.5 × 6.5 | II | IDC |
| Pat.#1027 | 367326 | B | 65 | 9 × 16 ×4.5 | 3.5 × 2.5 × 1.5 | II | IDC |
| Pat.#1028 | 123987 | C | 59 | 8.5 × 10 ×5 | 3.5 × 4 × 2.5 | III | IDC |
| Pat.#1029 | 192745 | D | 58 | 13 × 17 × 3. | 1.5 × 2.3 × 2 | I | IDC |
| Pat.#1030 | 267102 | C | 60 | 11 × 16 ×9.5 | 3.5 × 2.5 × 4.5 | II | IDC |
| - *Breast type according to the American College of Radiology (ACR) - Type A: Fatty Breast. - Type B: Scattered Volume Breast. - Type C: Heterogeneously Volume Breast - Type D: Extremely Volume Breast. | | | | - Grade I: Clearly distinguishable - Grade II: Moderately distinguishable - Grade III: Hard to be distinguishable - IDC: Invasive Ductal Carcinoma | | | |
